# Leisure time management mediates the relationship between job search anxiety and career planning

**DOI:** 10.1038/s41598-026-41054-9

**Published:** 2026-02-26

**Authors:** Sevim Handan Yilmaz, Gökhan Dokuzoğlu, Ali Çevik, Serdar Ceyhun

**Affiliations:** 1 Faculty of Sport Science, Bartın University University, Bartın, Turkey; 2 T.R. Ministry of National Education, Aydin Provincial Directorate of National Education, Aydın, Turkey; 3 Faculty of Sport Science, Kilis 7 Aralık University, Kilis, Turkey

**Keywords:** Sports sciences, Job search anxiety, Career planning, Leisure time management

## Abstract

This study aimed to examine the mediating role of leisure time management in the relationship between job search anxiety and career planning among students of the faculty of sports sciences. A total of 390 sports science faculty students participated in the study, 156 of whom were female and 234 were male. The questionnaire used to collect the relevant data consisted of four sections. The first section of the study included demographic information, the second section included the ‘Leisure Time Management Scale,’ the third section included the ‘Sports Science Students’ Job Search Anxiety Scale,’ and the fourth section included the ‘Career Planning Scale for Students Studying Sports Science.’ SPSS 25.0 and AMOS 21.0 software packages were used to analyze the data. The findings show a moderate, significant, and positive relationship between participants’ career planning and leisure time management; a weak, significant, positive relationship between career planning and job search anxiety; and a weak, significant, positive relationship between job search anxiety and leisure time management. The results revealed that job search anxiety has a significant effect on career planning and that leisure time management plays a mediating role in the impact of job search anxiety on career planning.

## Introduction

The transition from higher education to the labor market has become increasingly complex for today’s university graduates due to intensified competition, uncertainty in working conditions, and the limited availability of employment opportunities. These challenges contribute to the emergence of job search anxiety among higher education students during the post-graduation period^1^. Job search anxiety is defined as a psychological construct that plays a significant role in individuals’ career development, decision-making among career alternatives, and overall career planning processes^2^. For students receiving education in the field of sports sciences, this issue holds particular significance. The relatively limited employment opportunities available to sports sciences graduates, the demanding working conditions in the private sector, and ambiguities related to professional roles are among the factors that intensify job search anxiety within this population. Recent studies indicated that job search anxiety experienced by sports sciences graduates after graduation negatively affects their career planning processes^3,4^. Experiencing job search anxiety at high levels may cause to multiple challenges in individuals’ career planning. Career planning refers to the systematic process through which individuals identify their career goals in line with their skills, interests, and values, and develop strategies to achieve these goals. Research conducted on these issues indicates that increasing levels of job search anxiety are associated with decreased competencies in students’ career planning processes, an orientation toward more uncertain career paths, and elevated levels of career indecision^1,5^. Therefore, job search anxiety not only directly influences career planning but may also exert indirect effects through various psychological mechanisms. Thus, the behavioral and personal resources possessed by university students have gained increasing attention. Recent research in the field of career psychology emphasizes that individual resources such as time management, career adaptability, and self-regulation skills can mitigate anxiety experienced during the job search process^6,7^. One of these resources, leisure management, is examined as the ability of individuals to plan and use their leisure time purposefully. Leisure time management is not only associated with students’ academic achievement but is also closely linked to future planning, stress coping capacities, and psychological well-being. Current studies demonstrated that students with well-developed time or leisure management skills are more capable of maintaining a balance between academic and work-related responsibilities and exhibit higher life satisfaction, lower anxiety levels, and stronger career preparation behaviors^8–10^. Moreover, effective leisure time management may support the career planning process by increasing students’ participation in career-related activities such as professional development programs, internships, and certification courses^11,12^.

### Job search anxiety

Anxiety is an emotional state that arises when faced with an unexpected danger or any element that threatens the future. It emerges due to an individual’s differing perceptions of negative situations and the various meanings they assign to them^13^. An anxious person is in a constant state of fear, feels extremely uncomfortable, and is in a state of mental distress and obsession. The person worries that certain situations that do not exist may occur. Nevertheless, anxiety is a warning sign of danger, and people are constantly alert to this danger. It is a fundamental emotion that distinguishes humans from other living beings^14^.

Anxiety has recently become a widespread condition. It complicates human life and negatively impacts their quality of life. Environmental factors are among the most significant causes of this situation^15^. Individuals who live in their environment struggle to achieve specific goals, such as developing and enhancing their status within their family and society and assuming a primary identity. Individuals are part of a chain of fundamental criteria, such as starting a family, finding a job, gaining social status, and self-actualizing. One of the most important and indispensable parts of this chain is the anxiety that individuals experience regarding work and finding employment^16,17^. Job search anxiety is related to a stimulus, meaning that it arises in the workplace or when one is thinking about the workplace and is related to it. It is an important aspect of workplace life and significantly impacts well-being and health of employees^18^.

Job search anxiety encompasses various emotional, cognitive, and behavioral aspects experienced by individuals during the job search process^19^. Additionally, job search anxiety falls under the category of state anxiety, which is one of two types of anxiety, the other being trait anxiety^20^. Therefore, job search anxiety is shaped by environmental and family factors. Although it is often stated that job search anxiety is not experienced owing to the education individuals receive at school and their professional experience, this has not been possible recently^21,22^. One of the groups that experiences job search anxiety the most is university students. This is because university students may encounter many factors during their education process. This situation includes factors such as the city where education is received, socioeconomic level, security, and competition^23,24^. In addition, some factors affecting the anxiety experienced by students who are concerned about finding employment have been identified. These factors include feelings of pessimism, lack of self-confidence, the belief that one’s knowledge and skills are insufficient, social pressure from those around them, and declining employment rates, leading to increased unemployment^25^. Students do not believe that their education will be sufficient to find employment after graduation. For this reason, students prefer to participate in various courses, prepare for exams, or pursue master’s and doctoral degrees to develop their skills and abilities. On the other hand, male students first enlist in the military to increase their chances of finding employment^26^. In addition, the rise in educational levels and the increasing number of educated individuals are increasing the pressure young people face in finding employment^27^. In addition to high unemployment rates among young people and university graduates, the increase in the number of universities, the rise in the participation of university graduates in the workforce, and the perception of unemployment as a source of error, incompetence, and shame have contributed to the uncertainty and anxiety experienced by university students regarding their future and finding employment^28^. Most students spend much of their time searching for jobs on job search portals and the internet and seek counseling services regarding employment^2^. For these reasons, university students face many obstacles and difficulties in their career planning.

### Career planning

Career goals are a process that individuals must pursue within a framework of planning from their school years onward^29^. A career, in line with Maslow’s hierarchy of needs, refers to individuals progressing in their professional lives in a mentally healthy manner, aimed at self-realization, after fulfilling their physical needs, and reaching their desired destination^30^. A career is also defined as the totality of experiences that individuals gain throughout their lives^31,32^. Individuals strive to determine their attitudes toward their careers, their values, and the roles they play in life^33^. Career planning involves individuals discovering who they are, identifying their talents, evaluating opportunities around them, and exploring who they want to be and where they want to be in the future^34,35^. Career planning is important in determining identities and positions of individuals within society. Therefore, individuals view career planning as a social factor that provides them with the financial power necessary to sustain life. In addition, career planning is regarded as important in terms of psychological job satisfaction and personality development^36–38^. There are two main approaches to career planning: the organisational approach and the individual approach. The individual approach involves determining methods that suit their character structure. When these methods are used, the individual may be influenced by many factors. On the other hand, the organisational approach involves efforts and aims to combine the tendencies of individuals and goals with the organization’s goals in career-related planning^39^.

Recent studies on university students’ career planning and choices have revealed striking results. For example, students may be indecisive during the career planning process and may evaluate job opportunities in the market, not in the fields in which they have been educated but in other positions^40–42^. It is crucial to begin career planning during the education period to secure employment after graduation, develop skills relevant to the field, and adapt more easily to professional life^43^. The fact that students typically begin considering their careers only after graduation indicates that they lack sufficient awareness of this issue during their education. University years are a period when students should explore their careers. Guiding them to make the right choices at this stage is extremely important. Otherwise, students cannot plan the best way to enter a profession, which wastes time and resources while trying to achieve their career goals^44,45^.

Although it is difficult to predict who will be successful in the future workplace, it is not unreasonable to assume that people who plan their careers during their university years are more likely to be successful in the workplace than the others. This is because individuals can become aware of their talents and shortcomings through the career plans they make during their university years. With this awareness, a student’s education helps address professional shortcomings while also contributing to the development of positive qualities. Thus, when looking for a job or making career choices, individuals can seize the opportunity to pursue the best job instead of looking for a job randomly^46^. In addition, one of the points that university students should pay attention to when planning their careers is how they manage their leisure time.

### Leisure time management

In today’s world, the concept of time, which is constantly and rapidly evolving, has begun to gain importance daily^47^. Time is a valuable and unique resource that is equal for all individuals but cannot be used in the same way^48^. With technological developments and mechanization, individuals have gained free time. Free time is the process of voluntarily planning and organizing how to spend the time remaining after one’s basic needs are met^49–51^. Leisure time includes time not spent working, but not every situation characterized as leisure time is considered free. This is because free time is utilized by adapting non-work activities to leisure time^52,53^. In modern societies, evaluating free time is a matter that improves and increases people’s living standards and contributes to their self-discovery and renewal^54,55^. Moreover, making effective and efficient use of leisure time is important not only for increasing people’s work productivity but also for the emergence of a culturally creative community^56,57^. Furthermore, although leisure activities are considered important for a healthy life, the key to an active and healthy life is linked to managing leisure time^58,59^. These skills may vary depending on leisure activities, societal perceptions, and the environment and conditions in which they are experienced. Therefore, time management can manifest itself in various ways for individuals and societies^60^.

Leisure time management involves the process of planning, organizing, directing, coordinating, and supervising activities that people engage in to use their leisure time^61^. If individuals manage their free time well, they will participate more in recreational activities that contribute to their rest, enjoyment, and personal development^62^. These experiences or activities can increase the work performance of employees and reduce their stress while having a positive or negative effect on academic achievement, socialization, and career plans, especially among university students^63–65^.

Many people cannot make good use of their free time because of the fast pace of life and the desire to earn money. However, it is considered a basic necessity for all age groups. Additionally, it holds particular importance for university students transitioning from adolescence to adulthood and preparing for professional careers^66^. University students must use their free time effectively to relieve work stress and job search anxiety; develop new hobbies and life skills; regain their passion, hope, and enthusiasm for life; and plan their careers^67,68^.

Leisure time management has critical importance for students pursuing education in the field of sports sciences. In addition to their academic coursework, these students are required to engage in time-intensive activities such as practical sports classes, training sessions, and competitive events. Thus, students who are able to manage their leisure time effectively and efficiently are more likely to clarify their career goals and planning processes, enter the job search process with greater preparedness, and thereby alleviate their levels of job search anxiety. Within this framework, existing literature increasingly examines the relationships among job search anxiety, career planning, and time or leisure time management. However, the mediating role of leisure time management in the interaction between these variables has not yet been comprehensively investigated. Accordingly, the study aims to examine the effect of job search anxiety on career planning among students enrolled in faculties of sports sciences, to test the mediating role of leisure time management in this relationship, and to empirically and quantitatively model the relational structure among these three variables. By adopting a more holistic perspective on the career development processes of sports sciences students, this study seeks to highlight the importance of leisure time management in mitigating the negative effects of job search anxiety. It is expected that the findings will contribute to the academic literature in the field of sports sciences and shed light on the development of career counseling and intervention programs for students.

### Job search anxiety and leisure time management

Job search anxiety is conceptualized as an emotional and cognitive stress response to uncertainties in employment opportunities faced by university students following graduation. According to Stress Coping Theory, high levels of stress impair individuals’ cognitive functioning and negatively affect their self-regulation capacities. Recent empirical studies have demonstrated that job search anxiety weakens individuals’ self-regulatory abilities and adversely influences effective time management^69,70^. Moreover, high anxiety levels reduce individuals’ ability to plan and structure their leisure time, leading to increased tendencies toward procrastination and avoidance behaviors^71^. Research conducted particularly among university student populations indicated that anxiety serves as a significant predictor of leisure time management and time awareness^25,72^.

Considering the aforementioned theoretical and empirical links, a hypothesis was developed to explain the relationship between job search anxiety and leisure time management:

H1: There is a relationship between job search anxiety and leisure time management.

### Job search anxiety and career planning

Career planning is defined as the process through which individuals identify their professional goals and deliberately develop strategies to achieve these goals. Career Construction Theory emphasizes the importance of enhancing psychological adaptability and future orientation to enable individuals to actively construct their careers. Within the contemporary career planning literature, job search-related anxiety has been shown to negatively affect decision-making clarity and future orientation in career-related contexts^73,74^. Job search anxiety increases individuals’ perceptions of uncertainty, leading them to disengage from career planning goals and to postpone career-related decisions^75^. Recent studies conducted with university student samples indicated that high levels of job search anxiety significantly reduce career adaptability skills and career planning behaviors^5,76^.

Considering the theoretical and empirical relationships outlined above, a hypothesis was developed to explain the association between job search anxiety and career planning:

H2: There is a significant relationship between job search anxiety and career planning.

### Leisure time management and career planning

Leisure time management refers to individuals’ ability to deliberately plan and utilize their free time in ways that promote rest, recovery, and personal development. Conservation of Resources (COR) Theory conceptualizes time, energy, and psychological well-being as core personal resources and emphasizes that the effective management of these resources facilitates the attainment of long-term goals. Recent research demonstrated that effective leisure time management enhances self-efficacy, regulates stress, and positively influences goal-directed behaviors^77,78^. When concluded within the framework of Conservation of Resources Theory, the effective and efficient use of leisure time not only strengthens individuals’ cognitive and emotional resources but also facilitates career planning processes and the achievement of career-related goals^77,78^. Recent empirical studies have identified positive associations between leisure time management skills and occupational awareness, career decisiveness, and career planning behaviors^79,80^.

Considering the theoretical and empirical relationships outlined above, a hypothesis was developed to explain the association between leisure time management and career planning:

H3: There is a significant relationship between leisure time management and career planning.

### The mediating role of leisure time management

Recent literature about stress and career development indicated that the impact of job search anxiety on career planning is not solely direct but is also shaped through individuals’ psychosocial resources^75,81^. This relationship can be explained through several theoretical perspectives embedded within the leisure time management literature, most notably Boundary Theory and Psychological Detachment Theory. Boundary Theory focuses on how individuals organize and manage different life domains, emphasizing the balance between work/school and leisure. Within this framework, establishing a harmonious balance between work or academic demands and leisure time enables individuals to clarify life and career goals. Effective management of leisure time allows individuals to distance themselves from stressors originating in work or academic contexts, facilitates emotional and cognitive recovery, and promotes psychological rejuvenation^10,82,83^. On the other hands, Psychological Detachment Theory emphasizes the importance of drawing clear boundaries between work/school and leisure. According to this perspective, maintaining distinct separations between these domains prevents work- or school-related stress from spilling over into leisure time, thereby supporting overall well-being and functional recovery. For university students in particular, establishing clear distinctions between study periods and leisure time may enhance academic performance and contribute to more structured and coherent career planning processes^84–86^. The Social Psychology of Time Theory provides an additional theoretical framework for understanding this relationship by emphasizing the social and psychological dimensions of time use and its impact on individuals’ lives. Among young adults, such as university students, time management practices may vary depending on social interactions, career aspirations, personal development needs, and requirements for psychological recovery^87,88^. Moreover, effective leisure time management contributes to psychological restoration by reducing anxiety levels, thereby enabling individuals to allocate greater cognitive resources to career-related decision-making processes^78^. From this perspective, leisure time management functions as a mediating mechanism that attenuates the negative effects of job search anxiety on career planning. Recent empirical studies support this proposition by demonstrating that time and leisure time management skills play a significant mediating role in the relationship between stress and career-related outcomes^5,71^.

Based on these theoretical frameworks and empirical findings, the following hypothesis was formulated:

H4: Leisure time management plays a mediating role in the effect of job search anxiety on career planning.

## Methods

### Research Model

This study was designed within the framework of the basic mediation model to determine how sports science students’ job search anxiety affects their career planning through leisure time management. The basic mediation model is a path analysis that explains how the independent variable affects the dependent variable^89^. In this study, a survey research method, a quantitative analysis method, was applied. Correlational and causal survey techniques were used for the survey research. A correlational survey is a research model that determines the existence and/or degree of change between two or more variables^90^. In causal survey research, one or more independent variables that affect one or more dependent variables are identified, and generally, the antecedent variables that predict a dependent variable or the possible outcomes (consequences) of a variable are revealed, or both situations are examined together. In causal survey designs, the mediating variable effect between two variables is also investigated^91^.

**Fig. 1:**
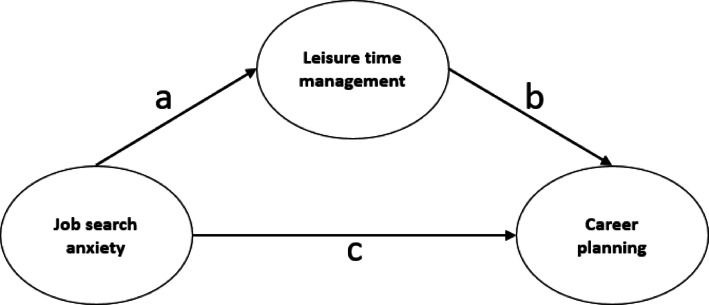
Basic mediation model.

As shown in Fig. 1, the direct effect of job search anxiety on leisure time management is indicated by a, the effect of leisure time management on career planning is indicated by b, and the effect of job search anxiety on career planning is indicated by c. The effect of leisure time management on job search anxiety on career planning (indirect effect) is indicated by c.

The following hypotheses were formulated based on this model:

H1: There is a relationship between job search anxiety and leisure time management.

H2: There is a relationship between job search anxiety and career planning.

H3: There is a relationship between leisure time management and career planning.

H4: Leisure time management plays a mediating role in the effect of job search anxiety on career planning.

### Participants

Convenience sampling was used to determine the population of this study. The study population consisted of 801 students enrolled at Bartın University Faculty of Sports Sciences during the 2024–2025 academic year, while the sample group consisted of 156 female and 234 male students, for a total of 390 students. Students enrolled in the Faculty of Sports Sciences were included in the study. The students participated in the study voluntarily. The students had the right to refuse to participate in the study or withdraw from any part of the study. The data were collected from the participants via Google Forms, and participation in the study was voluntary. The distribution of the study group according to demographic characteristics is presented in Table 1.

**Table 1:** Demographic variables.

Variables		f	%
Gender	Female	156	40.0
	Male	234	60.0
Grade Level	1st Grade	67	17.2
	2nd Grade	135	34.6
	3rd Grade	89	22.8
	4th Grade	99	25.4
Department	Teaching	94	36.3
	Management	32	12.4
	Coaching	27	10.4
	Recreation	40	15.4
Leisure Time Activity Type	Sports Activities	190	48.7
	Artistic Activities	29	7.4
	Reading Books	36	9.2
	Social Media-TV	86	22.1
	Other	49	12.6
Total		390	100

### Ethical approval

The Bartın University Social and Human Sciences Ethics Committee reviewed the study’s ethics committee at its meeting on 11 December 2024 and approved it with decision number 01 and protocol number 2024-SBB-0841. The study was conducted in accordance with the ethical principles outlined in the Declaration of Helsinki. All participants were fully informed about the process and purpose of the study, and written consent/assent was obtained.

### Data collection tools

The questionnaire used to collect the relevant data in the study consisted of four sections. The first section included demographic information, the second included the ‘Leisure Time Management Scale,’ the third included the ‘Sports Science Students’ Job Search Anxiety Scale,’ and the fourth included the ‘Career Planning Scale for Students Studying Sports Science.’

### Personal information form

The personal information form prepared by the researchers included questions about gender, class level, department, and leisure time evaluation methods.

### Leisure time management scale

The scale developed by Wang and colleagues (2011) and adapted into Turkish by Akgül and Karaküçük (2015) to measure the ‘Leisure Time Management’ levels of sports science faculty students is a 5-point Likert scale (1 = Strongly Agree, 5 = Strongly Disagree)^48,92^. It consists of 15 items and includes four subscales: goal setting and methods, leisure attitudes, programming, and evaluation. In the Turkish adaptation of the scale, the Lawshe technique was employed. For the adaptation study, the original scale, consisting of 4 sub-dimensions and 15 items, was firstly translated from its English version into Turkish and subsequently back-translated into English. After comparing the translated items with those of the original scale, necessary revisions were made, and the final Turkish version of the scale was obtained. The study group for the Turkish adaptation process consisted of 447 university students enrolled in various departments of Gazi University, Turkiye. The Cronbach’s alpha value for the Turkish adaptation of the scale was calculated as 0.83. The Cronbach’s alpha coefficient for this study was 0.863 (Table 2). The fit indices for the Turkish adaptation of the scale were calculated as χ2/df = 1.44; RMSEA = 0.056; SRMR = 0.076; CFI = 0.97; GFI = 0.90.

**Table 2:** Descriptive values related to scales.

Variables	Cronbach alpha	x̄	Ss	Skewness	Kurtosis
LTM	0.863	3.55	0.588	0.083	0.351
JSA	0.935	3.59	0.947	-0.349	-0.223
CP	0.946	3.79	0.613	-0.463	0.879

LTM, leisure time management; JSA, job search anxiety; CP, career planning.

### Employment anxiety scale for sports science students

The ‘Sports Science Students’ Job Search Anxiety Scale’ developed by Aslan and Uğraş (2021) to measure the job search anxiety levels of sports science faculty students is a 5-point Likert-type scale (1 = never true, 5 = usually true) consisting of 8 items and a single dimension^93^. During the scale development process, an initial item pool consisting of 19 items was generated. Following the creation of the item pool, the items were sent to 5 faculty members with expertise in Physical Education and Sports Teaching and Sport Psychology to evaluate the content validity of the items. The study group for the scale development process consisted of 525 students enrolled in various departments of the Faculty of Sport Sciences at Çanakkale Onsekiz Mart University, Turkiye. Subsequently, as a result of the validity and reliability analyses conducted on the scale, an 8-item unidimensional structure was obtained. During the development process of the scale, the Cronbach’s alpha value was calculated as 0.958. The Cronbach’s alpha coefficient for this study was calculated as 0.935 (Table 2). During the development process of the scale, the fit indices were calculated as χ2/df = 3.257; RMSEA = 0.08; CFI = 0.98; GFI = 0.95.

### Career planning scale for students studying sports science

The ‘Career Planning Scale for Students Studying Sports Science’ developed by Eroglu and Eroglu (2020) to measure the career planning levels of sports science faculty students is a 5-point Likert scale (1 = Strongly Agree, 5 = Strongly Disagree) that consists of 23 items and five subscales: career awareness, professional awareness, career-related beliefs, accuracy of choice, and adequacy of education. During the scale development process, an initial item pool consisting of 35 items was generated. Following the creation of the item pool, the items were reviewed by five experts in the field of Physical Education and Sport and three experts in the field of Economics and Administrative Sciences to assess the content validity of the items. The study group for the scale development process consisted of 543 students enrolled in various departments of the Faculty of Sport Sciences at Siirt University, Turkiye. Based on expert evaluations, 5 items were removed, and a 30-item pilot form was created. Subsequently, as a result of the validity and reliability analyses conducted on the scale, a 23-item structure with five dimensions was obtained. The Cronbach’s alpha value was calculated as 0.88 during the scale development process^94^. The Cronbach’s alpha coefficient for this study was 0.946 (Table 2). During the development process of the scale, the fit indices were calculated as χ2/df = 2.42; RMSEA = 0.05; CFI = 0.92; GFI = 0.92.

### Data analysis

The data obtained in the study were analyzed via the SPSS 25.0 and AMOS 21.0 software packages. The reliability of the data collection tools was determined via Cronbach’s alpha. The skewness and kurtosis coefficients were examined in the data distribution, and the data were distributed between ± 2, indicating that the data were normally distributed^95^. The Pearson correlation test was applied to determine the relationships between the dependent and independent variables. Confirmatory factor analysis was performed to determine the validity of the measurement tools. Path analysis was performed to determine the level of influence between the variables. A 5% margin of error was used as a reference in the study.

## Results

In Table 1, the variables with the highest percentages of data obtained from the participants are male sex (60.0%), 2nd-class status (34.6%), physical education and sports department status (36.3%), and the free-time evaluation method variable in participants who selected the sports activity option (48.7%).

Table 2 shows the Cronbach’s alpha coefficients, mean scores, standard deviations, kurtosis values and skewness values for the scales.

Table 3 shows a moderately significant and positive relationship between participants’ career planning and leisure time management (r = 0.648). A weak, significant, and positive relationship exists between career planning and job search anxiety (r = 0.214). and a weak, significant, and positive relationship between job search anxiety and leisure time management (r = 0.128).

**Table 3:** Results of pearson correlation analysis between variables.

Dimensions	1-	2-	3-
1-LTM	1		
	–		
2-JSA	0.128*	1-	
	0.000		
3-CP	0.648**	0.214**	1
	0.000	0.000	–

*p* < 0.01**. *p* < 0.05* LTM, leisure time management; JSA, job search anxiety; CP, career planning.

Table 4 showed confirmatory factor analyses of scales of leisure time management, job search anxiety and career planning. Confirmatory factor analysis was performed for construction validity of the scales.

**Table 4:** Findings related to confirmatory factor analyses.

	χ2/df	RMSEA	CFI	GFI	TLI	SRMR
LTM	3.493	0.079	0.915	0.917	0.892	0.0605
JSA	2.920	0.070	0.985	0.966	0.977	0.0224
CP	3.239	0.076	0.903	0.859	0.888	0.0492

LTM, leisure time management; JSA, job search anxiety; CP, career planning χ2/df ≤ 5; RMSEA ≤ 0.08; GFI ≥ 0.85; SRMR ≤ 0.08^96^; CFI ≥ 0.80^97^; TLI ≥ 0.80^98,99^.

**Fig. 2:**
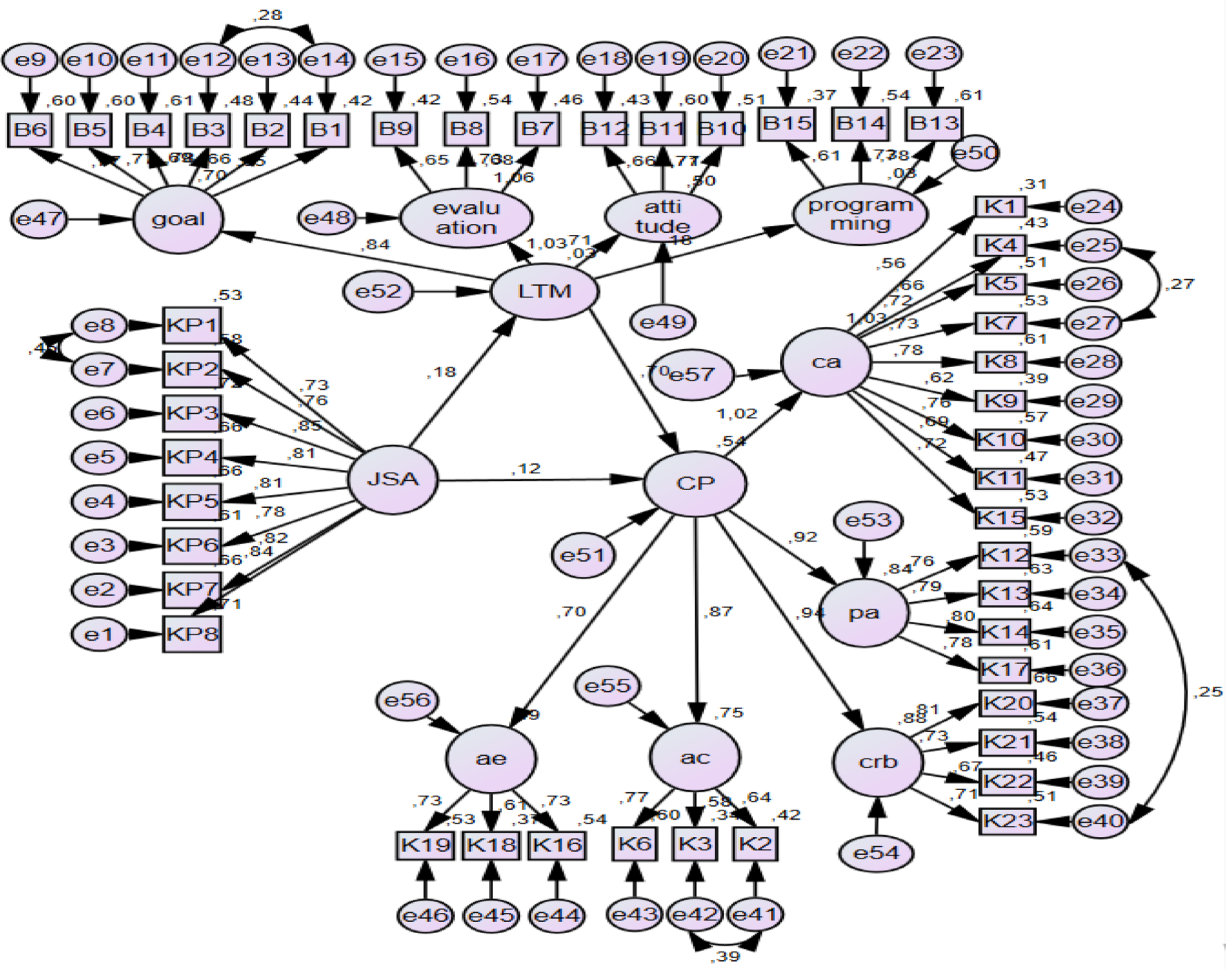
Path diagram related to the mediating role of leisure time management in the effect of job search anxiety on career planning.

A model was created to test whether leisure time management plays a mediating role in the effect of job search anxiety on career planning as shown in Fig. 2 and Table 5.

**Table 5:** Results of the model on the mediating role of leisure time management in the effect of job search anxiety on career planning.

Direct effect		St. Est	S.E	CR		*p*
JSA → LTM		0.177	0.038	3.068		0.002
LTM → CP		0.703	0.074	8.499		***
JSA → CP		0.116	0.025	2.71 +		0.007
**Total Effect**	**St. Est**	**Lover**	**Upper**	**S.E**	**p**	
JSA → CP	0.241	0.101	0.372	0.069	0.002	***
**Indirect Effect**	**St. Est**	**Lover**	**Upper**	**S.E**	**p**	
JSA → LTM → CP	0.125	0.015	0.242	0.058	0.027	***
(χ2/df = 2.367; RMSEA = 0.059; SRMR = 0.0702; CFI = 0.875; GFI = 0.812; TLI = 0.867)						

*p < 0.05 LTM, leisure time management; JSA, job search anxiety; CP, career planning.

In the created model, the effect of the independent variable on the dependent variable was examined before the mediating role was explored. Job search anxiety was observed to have a statistically significant effect on career planning without a mediator (β = 0.241, *p* < 0.05). The effect of the independent variable on the dependent variable was significant. The presence of a mediating role in this effect was examined, according to the results obtained from the model. The fact that the 95% confidence interval values do not include 0 indicates that there is a mediating role in the model (0.015, 0.242) after deciding that there was a mediating role. The significance of the direct effect was examined to determine the type of mediating role. It was concluded that the direct effect was significant. However, the effect coefficient value decreased (β = 0.125, *p* < 0.05), and it was decided that the mediator was a partial mediator. Because the goodness-of-fit values in the applied model were not acceptable, some modifications were made to the residuals after the modifications. The goodness-of-fit values of the model were examined. Moreover, all of them were at an acceptable level (χ^2^/df; RMSEA = 0.042; SRMR; CFI = 0.966; GFI = 0.912; TLI = 0.963).

## Discussion

This research was conducted via a survey model and aimed to determine the examine the mediating role of leisure time management in the relationship between job search anxiety and career planning among students of the faculty of sports sciences. In this context, the mediating role of leisure time orientation in the relationship between job search anxiety and career planning among Faculty of Sports Sciences students was examined. A total of 390 students participated in the study. A total of 234 patients were male, and 156 were female. The demographic distribution of the participants was examined. The number of male participants in the study was greater than that of female participants, and most were students from physical education and sports departments. The participants’ leisure time evaluation methods were examined. They preferred sports activities, and most participants tended to spend their leisure time doing sports activities. This situation may be because the study was conducted in the faculty of sports sciences. However, the young population in particular needs to spend their free time meaning fully to prevent sociocultural, economic and educational problems^100^.

Within the scope of the research, the results of the Pearson correlation analysis between job search anxiety leisure time management variables were examined. There is a very low but significant and positive relationship between leisure time management and job search anxiety. This finding indicates that leisure time management does not directly affect job search anxiety but still has an indirect effect. In this case, individuals who manage their free time well may experience more job search anxiety, albeit at a very low level. Although this situation may seem complex, these individuals place greater importance on career planning because they consciously evaluate their free time, which may increase their job search anxiety. While effective leisure time management increases an individual’s capacity to cope with stress, it also makes it difficult to completely control factors such as job search anxiety^61^. Recent studies indicated that university students’ job search anxiety affects not only stress coping mechanisms and psychological well-being but also their approaches to time and leisure time management and planning. In a study conducted with university students, Han (2022) identified a significant negative relationship between time management tendencies and job search anxiety, emphasizing that students with weaker time management skills experienced more intense anxiety during the job search process, which in turn disrupted their daily time use patterns^101^. Similarly, Dong et al. (2023) reported that time management skills are closely associated with stress and anxiety levels, noting that as anxiety increases, individuals’ tendency to use their time in a purposeful and planned manner decreases^102^. In another study, Yazıcı et al. (2023) found that higher levels of job search anxiety negatively affected life satisfaction, whereas greater engagement in leisure crafts contributed positively to life satisfaction^103^. When interpreted in the context of students enrolled in sports sciences programs, these findings gain particular relevance. In addition to academic responsibilities, sports sciences students are required to engage in time-intensive commitments such as training sessions, competitions, and various physical activities. As a result, these students may be more likely to allocate their leisure time to passive activities driven by anxiety rather than to restorative, career-oriented, or personal development activities. This pattern may enhance their levels of job search anxiety. Our study, in parallel with the literature demonstrated meaningful association between job search anxiety and leisure time management, thereby providing empirical support for Hypothesis 1.

Within the scope of the research, the results of the Pearson correlation analysis between job search anxiety and career planning variables were examined. Thus, there is a weak but significant and positive relationship between career planning and job search anxiety. Job search anxiety is observed in participants who engage in career planning even at a weak level ın this case. Importantly, regardless of how well an individual plans their career, they cannot completely escape anxiety due to existing uncertainties and the competitive environment. In the field of higher education, the issues of job search anxiety and career planning have been extensively investigated in recent years. Because job-seeking anxiety is linked to people’s fears of not being able to find a job, not being able to achieve economic independence, and not being able to attain a professional status. Such concerns may intensify students’ levels of job search anxiety and, consequently, hinder the development and realization of long-term career plans and goals^5^. Studies in the literature show that job search anxiety affects career planning processes^104,105^. Thus, job search anxiety can influence an individual’s career planning, determination, and strategy development. While job search anxiety may cause individuals to plan their careers better the opposite may also be true. In their research, Akdemir (2021) and Abacıoğlu (2019) examined university students and job search anxiety and reported no significant difference between job search anxiety and the departments of university students they were studying in studies in the literature^106,107^, revealing that university students largely experience job search anxiety, especially after graduation, when they believe they will not be able to find a job^108^. Therefore, it should be noted that career planning can motivate students but does not eliminate job search anxiety. However, engaging in career planning can help students reduce their job search anxiety to a more manageable level. In a study that investigated job search intentions of university students, Deer, Gohn & Kanaya (2018) examined the effects of anxiety on job search and career planning^109^. The results of the study suggest that low levels of anxiety have a positive effect on career planning and job search duration. Therefore, anxiety should have a weak mediating effect on the career planning process and job search intentions. Yılmaz and Caz (2021) reported that university students studying at the faculty of sports sciences have high levels of career planning and job search anxiety^22^. However, the relationship and direction between career planning and job search anxiety were evaluated in this study. No significant difference was observed, and it was determined that career planning and job search anxiety do not affect each other. In a study conducted by Arbona et al. (2021), the mediating role of anxiety in the relationship between intolerance of uncertainty and rumination and the three dimensions of career decision-making difficulties among university students was examined. The findings revealed that intolerance of uncertainty was both directly and indirectly (through anxiety) associated with all three dimensions of career decision-making difficulties, namely lack of readiness, lack of information, and inconsistent information^110^. Yazgan, Şendoğdu & Karadağ (2022) argued that individuals with high job search anxiety experience career uncertainty, which negatively affected their career planning^111^. In another study, Elfina and Weißmann (2025) empirically examined the dual effects of career planning education on reducing career anxiety and enhancing career self-efficacy among vocational high school students. Their findings demonstrated that career planning education constitutes a targeted strategy for strengthening psychological preparedness and decision-making skills among students facing uncertain career pathways^112^. Similarly, a study conducted by Kwak, Kim and Chae (2025) with university students reported that job search anxiety exerted negative effects on career attitudes and psychological well-being. In addition, students experiencing high levels of job search anxiety reported greater career indecision, lower motivation, and increased pessimism regarding their future career prospects^1^. From the perspective of students enrolled in sports sciences programs, the perceived limitation of post-graduation employment opportunities may further intensify job search anxiety, thereby negatively influencing attitudes toward career planning. In particular, students with elevated anxiety levels may be more likely to postpone career planning activities or engage in them in an unstructured and unsystematic manner. In this context, both the findings of the present study and the international literature demonstrated a significant relationship between job search anxiety and career planning, thereby providing empirical support for Hypothesis 2.

Within the scope of the research, the results of the Pearson correlation analysis between leisure time management and career planning variables were examined. There was a moderate positive relationship between the participants’ leisure time management and career planning. This situation shows that the more the participants plan their career planning, the more efficiently and consciously they use their leisure for management in this sense. The participants who think about and plan their future tend to evaluate their free time similarly. Recent studies suggested that time and leisure management skills are associated with individuals’ long-term planning behaviors, goal setting, and self-regulation^9^. Altuntaş et al. (2021) examined the relationships between university students’ time management and planning skills and their leisure satisfaction^113^. The study revealed that university students who can manage their time well and plan their lives well have good leisure satisfaction in this regard. The ability of students to manage their career planning processes has positive effects not only on their work life but also on their life order and goal achievement. Dong et al. (2023) reported that time management skills play a decisive role in academic achievement and career adaptability. Their study demonstrated that students who use their time effectively define their career goals more clearly and participate more actively in career planning processes. This finding indicated that leisure time management constitutes a personal resource that supports career planning^102^. In his research, Terzi et al. (2024) argued that individuals focusing on career planning are more successful in terms of time management productivity and personal development. In addition, the research revealed a positive relationship between university students’ free time management skills and their quality of life. The research concluded that students who can effectively manage their free time are task oriented and do not have problems meeting deadlines. In this sense, career planning suggests that students who perform well and work regularly should be supported in managing their free time. Moreover reported that individuals who have developed leisure time management are more conscious of career planning^10^. The conscious evaluation of leisure time management can strengthen career planning. Hunt (2025) also emphasized the importance of leisure time management for career planning, arguing that individuals with effective leisure time management place greater importance on career planning, increasing their expectations and self-efficacy^114^. The study conducted by Bilgin (2025) aimed to investigate the effects of nursing students’ attitudes toward leisure and leisure time management on their academic performance. According to the findings, travel/exploration of new places, gender, and participation in sports activities were identified as significant determinants of academic achievement among nursing students. A significant difference was found between nursing students’ overall academic classification and their scores on leisure time management, leisure attitudes, and technical sub-dimensions. Moreover, nursing students who managed their leisure time effectively, exhibited positive attitudes toward leisure, and consciously planned their leisure activities achieved higher academic success^115^. From the perspective of sports sciences students, leisure time management offers not only opportunities for rest and recreation but also significant advantages in terms of career development. Participation in certificate programs, involvement in voluntary sports organizations, and engagement in activities aimed at developing professional skills can be facilitated through effective leisure time management. Therefore, it is expected that sports sciences students who use their leisure time in a planned and purposeful manner will demonstrate higher levels of career planning. The findings of the present study, together with those reported in the literature, support the existence of a relationship between leisure time management and career planning, thereby confirming Hypothesis H3.

When the mediating role of leisure time management in the effect of career planning on job search anxiety was examined, it was observed that career planning significantly affects job search anxiety. Indeed, while career planning can support individuals in controlling their job search, it can also enable them to proceed more carefully in the job search process. Hypothesis H4, which constitutes the main hypothesis of the present study, posits that leisure time management partially mediates the effect of job search anxiety on career planning. Although studies in the literature that directly examine leisure time management as a mediating variable are limited, research conducted on similar psychological and behavioral variables provides theoretical support for this hypothesis. Indeed, increased anxiety about the future in individuals experiencing job search anxiety can lead to distractibility, tension, and irregular sleep patterns. In this context, career planning plays an important role in individuals’ management of employment anxiety. Individuals experience anxiety, especially when they lack information about the future and cannot control it^116^. Within the framework of this information, Yağmur and Ocak (2013) concluded in their study that leisure time management positively affects academic performance by increasing students’ study efficiency and that students with leisure time management feel mentally and physically well and satisfied^117^. The study conducted by Wang (2019) demonstrated that leisure time management reduces leisure boredom, while leisure boredom increases internet addiction. Furthermore, the findings revealed that leisure boredom plays a mediating role in the relationship between leisure time management and internet addiction^118^. In another study, Yuncu et al. (2020) aimed to examine the mediating role of leisure time management in the relationship between coping strategies for stress and leisure satisfaction. The findings indicated positive relationships among coping strategies, leisure time management, and leisure satisfaction, and further revealed that leisure time management partially mediated the effect of coping strategies on leisure satisfaction^119^. Similarly, Çerez et al. (2021) found positive linear relationships between leisure time management and participation in leisure-time exercise, between leisure time management and psychological well-being, and between leisure-time exercise participation and psychological well-being. The authors concluded that individuals who manage their leisure time effectively exhibit higher levels of leisure-time exercise participation and psychological well-being^120^. In another study, Terzi et al. (2024) investigated the mediating role of leisure satisfaction in the relationship between leisure time management and quality of life among university students. The results demonstrated significant relationships among leisure time management, leisure satisfaction, and quality of life. Moreover, leisure satisfaction was found to partially mediate the relationship between leisure time management and quality of life, with students’ ability to manage their time effectively emerging as a key factor in this relationship^10^. Furthermore, Harada et al. (2024) demonstrated that leisure time management is a significant determinant of subjective well-being among older adults, indicating that effective leisure time management enhances individuals’ subjective well-being^121^. Accordingly, time and leisure management skills strengthen students’ coping strategies for anxiety and enhance their perceived sense of control^9^. This mechanism may help mitigate the negative impact of job search anxiety on career planning. For students in the field of sport sciences, leisure time management reduces anxiety through physical activity, social interaction, and professional development activities, thereby providing a healthier foundation for career planning. In this context, leisure time management can be regarded as an important tool for maintaining a work–study balance, conceptualized as having sufficient time to fulfill responsibilities both during leisure and academic/work-related activities^8^.

The results of the present study, which are consistent with the existing literature, indicated that leisure time management partially mediates the relationship between job search anxiety and career planning. Specifically, the mediation analysis demonstrated a significant indirect effect of job search anxiety on career planning through leisure time management, as confirmed by bootstrap confidence intervals that did not include zero, while the direct effect remained statistically significant. The issue suggests a partial mediation model, indicating that leisure time management serves as an important, yet not solely sufficient, explanatory mechanism in this relationship. From a theoretical perspective, effective time and leisure management may enhance individuals’ perceived control, strengthen anxiety coping strategies, and support adaptive self-regulatory processes, thereby attenuating the negative impact of job search anxiety on career-related planning behaviors. For students in sport sciences, leisure time management may be particularly important, as engagement in physical activity, social interaction, and professional development activities during leisure time can reduce anxiety levels and foster a more structured and proactive approach to career planning. In this context, leisure time management may also contribute to maintaining a healthier work–study balance, enabling students to allocate sufficient time to both academic responsibilities and restorative or developmental leisure activities. Taken together, our studies as indicated previous literature suggested that leisure time management may play a partial mediating role in the relationship between job search anxiety and career planning. In this respect, Hypothesis H4 is supported both theoretically and empirically.

## Conclusions

In conclusion, the mediating role of leisure time management in the effect of sports science faculty students on career planning and job search anxiety was examined. As students’ career planning levels increase, they tend to evaluate leisure time management more efficiently; individuals who can develop career planning can increase their job search anxiety to a controllable level, and students who consciously evaluate leisure time management attach more importance to career planning. In addition, leisure time management plays a partial mediating role in terms of the effect of career planning on job search anxiety. Students who evaluate leisure time management in a planned manner perform career planning more consciously, and this situation can help them reduce their job search anxiety. Students’ development of career planning and leisure time management can contribute to their ability to manage job search anxiety. At this point, career planning reduces job search anxiety. Leisure time management is important, and it can be said that students who are effective in leisure time management perform career planning more consciously and can manage job search anxiety. In this context, students must invest in career planning and personal and social development. In addition, it is suggested that students with career planning should be provided with counseling services for both career planning and leisure time management, as students who perform leisure time management in a more planned manner can minimize their anxiety. Therefore, the research concludes that developing different programs that enable students to evaluate their leisure time activities, reduce their anxiety levels, and take more qualified steps in their leisure time activities is recommended. This approach is believed to support the multidimensional development of students.

Despite these contributions, some limitations must also be considered. The first, the cross-sectional design of the study restricts causal inferences regarding the directionality of the observed relationships. Although mediation analysis provides insights into potential explanatory mechanisms, longitudinal or experimental designs are needed to establish temporal precedence and causal pathways more robustly. The second, the reliance on self-report measures may introduce common method bias, which future studies could address by incorporating multi-source data or objective indicators of time use. The third, the reason that participants predominantly prefer sports activities as leisure activities may be that the study was conducted with students from the Faculty of Sports Sciences, which is one of the study’s limitations. In this context, it is recommended that the study be conducted with a broader and more diverse group of participants.

## Data Availability

The datasets used and/or analysed during the current study available from the corresponding author on reasonable request.
